# Surgery of the Nerves

**Published:** 1895-10-05

**Authors:** 


### SURGERY OF THE NERVES.

**Intra Cranial Neurectomy of the Trigeminus.**—Tiffany[1] supplements his previous paper[2] on four cases of this operation by adding other three. All the seven recovered, and, so far as he can learn, there has been no recurrence of the pain in any. From a study of his own cases he concludes that the middle meningeal artery is not a serious danger in the operation. Krause[3] agrees with him in this, although Konig[3] reports a case of fatal hæmorrhage from this artery. Tiffany considers it an advantage to open the dura to allow of the escape of some cerebro-spinal fluid, but Krause tries to avoid this. Chloroform is preferred to ether as causing less turgescence of the vessels of the head and face. The resulting anæsthesia and interference with thermic sense vary in extent, degree, and duration, as also does the interference with taste. Tiffany is not certain if the Gasserian ganglion is the *fons et origo mali*, and he does not always remove it. Krause resects the ganglion in preference to dividing the branches beyond, and in the two cases he reports not only had the pain been abolished, but no keratitis or other trophic disturbances had ensued two years later.

**Spinal Accessory Nerve.**—Dr. Elsworth Eliot[4] contributes an interesting paper having special reference to the spinal accessory nerve in the treatment of torticollis. In dealing with the surgical anatomy of torticollis the writer emphasises the importance of bearing in mind not only the abnormal condition of the sterno-mastoid muscle, but also the secondary changes which take place in other spinal and neck muscles, thus rendering the muscular element of torticollis a very complex one. In addition, fascial thickenings and contractions, and altered articular surfaces have also to be reckoned with. In spasmodic torticollis, he says, "as yet no localised inflammatory condition situated in that part of the central nervous system and upper spinal cord from which the spinal accessory and motor filament of the cervical plexus take origin has been established as a cause of this condition. In the treatment of congenital cases the first indication is to lengthen the short sterno-mastoid muscle by tenotomy of its sterno-clavicular attachment. The question most at issue at present is, whether the subcutaneous tenotomy is to be adhered to in preference to the open method. The tendency of surgical opinion seems to be in favour of the open method, which, with due aseptic precautions, gives greater certainty of dividing offending structures and less risk of damaging others.

**Spasmodic torticollis** requires something more than tenotomy for its cure. The reflex circuit must be broken. The spinal accessory nerves must be dealt with. The incision along the posterior border of the sterno-mastoid has the great disadvantage that the nerve is not easily recognised on account of its small size and its proximity to other nerves lying parallel with it, *i.e.*, the descending branches of the cervical plexus to the trapezius. This incision, however, entails very little risk to important vessels and nerves. A preferable incision is made along the anterior border of the sterno-mastoid—from the mastoid process to a point one or two inches below the angle of the jaw. The anterior edge of the muscle being exposed, the lower portion of the parotid gland may require to be pulled forward. The spinal accessory nerve is found to run downwards and forwards just below the transverse process of the atlas, which is easily felt. By keeping superficial to the posterior belly of the digastric the main vessels of the neck are avoided. Owen and Petit state that the nerve enters the muscle exactly opposite the angle of the jaw, but Eliot considers the transverse process of the atlas a better guide. The nerve lies a fraction of an inch in front of the most prominent part of that bone, and may easily be detected by drawing back the edge of the sterno-mastoid, when it stands out in relief among connective tissue. Having secured the nerve it may be stretched, but the good results of this procedure are short-lived. Better is it to resect from one-third to one inch of the nerve, and so produce complete paralysis of the sterno-mastoid and trapezius.

When the torticollis is kept up by spasmodic contraction of the deep posterior muscles, the best treatment is to divide and excise the posterior nerve roots as they issue from the intervertebral foramina. Smith has had a success with this operation, after stretching the spinal accessory failed.

In very aggravated cases, of long standing, it may be necessary to excise bodily a portion of contracted fascia or contracted deep muscles.

In all cases the operation must be supplemented by subsequent orthopædic treatment with suitable apparatus.

In a case of spasmodic torticollis Weiss[4] has used curare with apparent success. The patient suffered from torticollis, with spasms of the right sterno-mastoid and rotators of the head and vertebral column. Ordinary medicinal treatment and nerve-stretching had failed, and in three weeks after the operation half a Pravaz syringeful of curare (2·5 gr. to 167 gr. of water). This was repeated every second day, the dose being gradually increased till tremulousness was produced. A cure was effected.

**Dislocation of Ulnar Nerve at the Elbow.**—Wharton[5] contributes a paper on this subject, based on one case of his own and thirteen others which he has collected. Trauma was the cause in most of the cases. In a few, reduction by manipulation with a pad and splint were sufficient for treatment; in others, the nerve had to be stitched into position. In some cases resection was called for to remove pain and restore function.

